# The intersection of *Helicobacter pylori* and gastric cancer: signaling pathways and molecular mechanisms

**DOI:** 10.3389/fcimb.2025.1601501

**Published:** 2025-06-27

**Authors:** Zekun He, Yanan Zhou, Jianping Liu, Nianshuang Li, Huizhen Fan

**Affiliations:** ^1^ Department of Gastroenterology, Yichun People's Hospital, Jiangxi Medical College, Nanchang University, Jiangxi, China; ^2^ Department of Gastroenterology, Jiangxi Provincial Key Laboratory Project of Digestive Disease, Jiangxi Clinical Research Center for Gastroenterology, Digestive Disease Hospital, The First Affiliated Hospital, Jiangxi Medical College, Nanchang University, Jiangxi, Nanchang, China

**Keywords:** H. pylori, gastric cancer, signaling pathways, molecular mechanisms, gastric inflammation

## Abstract

*Helicobacter pylori* (*H. pylori*) is widely recognized as a potent risk factor for gastric adenocarcinoma, although only a small percentage of infected individuals develop malignancy. Recent advances have provided insights into how *H. pylori* contributes to gastric tumorigenesis through the modulation of inflammation, DNA damage, and cellular junctions via shared host cell targets and signaling pathways. A thorough examination of the signaling pathways altered by *H. pylori* infection could facilitate the discovery of previously unidentified infectious causes of cancer. This, in turn, would support the development of preventive strategies for *H. pylori*-related gastric malignancies by understanding the molecular mechanisms underlying pathogenesis. This review highlights recent advancements in understanding how *H. pylori* influences host cell signaling pathways to impact inflammation, genomic stability, abnormal cell proliferation, and other biological processes that promote the onset and progression of gastric cancer.

## 1 Introduction

While there has been a decline in the incidence and mortality rates of gastric adenocarcinoma in recent years, it continues to rank as the fourth most common cause of cancer-related deaths worldwide (Sung et al., 2021). It is worth noting that significant geographical variations exist in the occurrence and mortality patterns of gastric cancer (GC), with the highest incidence rates observed in Eastern Asian countries like Japan, Korea, and China (Torre et al., 2016; Thrift and El-Serag, 2020; Sung et al., 2021). The predominant form of gastric adenocarcinoma, known as intestinal-type gastric cancer, follows a sequential progression through various pathological stages. This evolution typically starts with non-atrophic gastritis, advances to atrophic gastritis, then to intestinal metaplasia, and ultimately progresses to dysplasia and malignant transformation.

*Helicobacter pylori*, a bacterium that colonizes the gastric pylorus, is recognized as the primary risk factor for gastric carcinoma. Multiple clinical studies indicate that eradicating *H. pylori* significantly reduces the risk of gastric cancer in infected individuals without precancerous lesions (Liou et al., 2020; Huang et al., 2023). Typically acquired early in life, often during childhood, *H. pylori* infections tend to persist throughout the host’s lifetime if left untreated. While gastritis commonly occurs in all individuals with *H. pylori* infection, only a small subset of those infected go on to develop gastric cancer (Salvatori et al., 2023). Variability in host response to specific virulence factors, influenced by genetic diversity, may underlie the varying clinical outcomes among *H. pylori*-infected individuals.

Among the identified virulence factors, cytotoxin-associated gene A (CagA) stands out as particularly relevant to the pathogenesis of *H. pylori* infection (Sedarat and Taylor-Robinson, 2024). Toll-like receptors activated by *H. pylori* induce pro-inflammatory responses, while the cag pathogenicity island-encoded type IV secretion system (T4SS) facilitates the transmission of pro-inflammatory molecules, leading to changes in pathological stages (Pachathundikandi et al., 2023). Evidence suggests that *H. pylori* stimulates cell inflammation and hyperproliferation, thereby promoting tumorigenesis by manipulating host cellular signaling pathways. This review aims to summarize recent progress in understanding the molecular mechanisms through which *H. pylori* infection exacerbates gastric carcinogenesis via the modulation of host cellular signaling pathways.

## 2 Key host signaling pathways dysregulated by *H. pylori* infection

Regulated signaling pathways play a crucial role in modulating various biological responses such as immune reaction, inflammation, cell proliferation, and cell survival. Emerging research indicates that *H. pylori* infection has the capability to manipulate these pathways to promote bacterial infection and cellular transformation.

### 2.1 NF-κB signaling

The Nuclear Factor-kappa B (NF-κB) signaling pathway consists of five members: p65 (RelA), RelB, c-Rel/Rel, p50 (NF-κB1), and p52 (NF-κB2), which can form homo- or heterodimers (Hoesel and Schmid, 2013). There are two recognized NF-κB pathways - the canonical and non-canonical pathways. The NF-κB pathway is notably significant in chronic gastritis (Lee et al., 2005; Zhu et al., 2017; Maubach et al., 2022), especially concerning the response to *H. pylori* infection (Guan et al., 2024).

Following the delivery of CagA into host cells by *H. pylori* via the T4SS, the IκB kinase (IKK) is activated, leading to phosphorylation and subsequent degradation of IκBα. This process results in the release of the NF-κB dimer, which translocates to the nucleus and modulates the expression of host genes, initiating downstream gene transcription (Mao et al., 2021). Activation of NF-κB through this pathway triggers the secretion of pro-inflammatory cytokines such as TNF-α, IL-1β, IL-6, and IL-8, along with the regulation of chemokine responses. These cytokines and chemokines can further activate the IKK complex by binding to their respective receptors, thereby amplifying the activation of the NF-κB signaling pathway (Chen et al., 2023; Martinelli et al., 2023).

Recent research has unveiled that ADP-heptose, a novel pathogen-associated molecular pattern (PAMP) involved in lipopolysaccharide (LPS) synthesis by *H. pylori* (Garcia-Weber and Arrieumerlou, 2021), acts as an antigen during *H. pylori* infection and triggers the activation of the NF-κB signaling pathway (Gaudet et al., 2015; Pfannkuch et al., 2019). This activation leads to the release of pro-inflammatory cytokines by stimulating the ADP-heptose/(α-kinase 1)-TIFA (TRAF-interacting protein with forkhead-associated domain) axis (Zhou et al., 2018; Garcia-Weber and Arrieumerlou, 2021). Intriguingly, studies by Naumann et al. have demonstrated that TIFA interacts with various E3 ubiquitin ligases, such as tumor necrosis factor receptor-associated factor (TRAF), to activate both canonical and non-canonical NF-κB pathways (Maubach et al., 2021).

**Figure 1:**
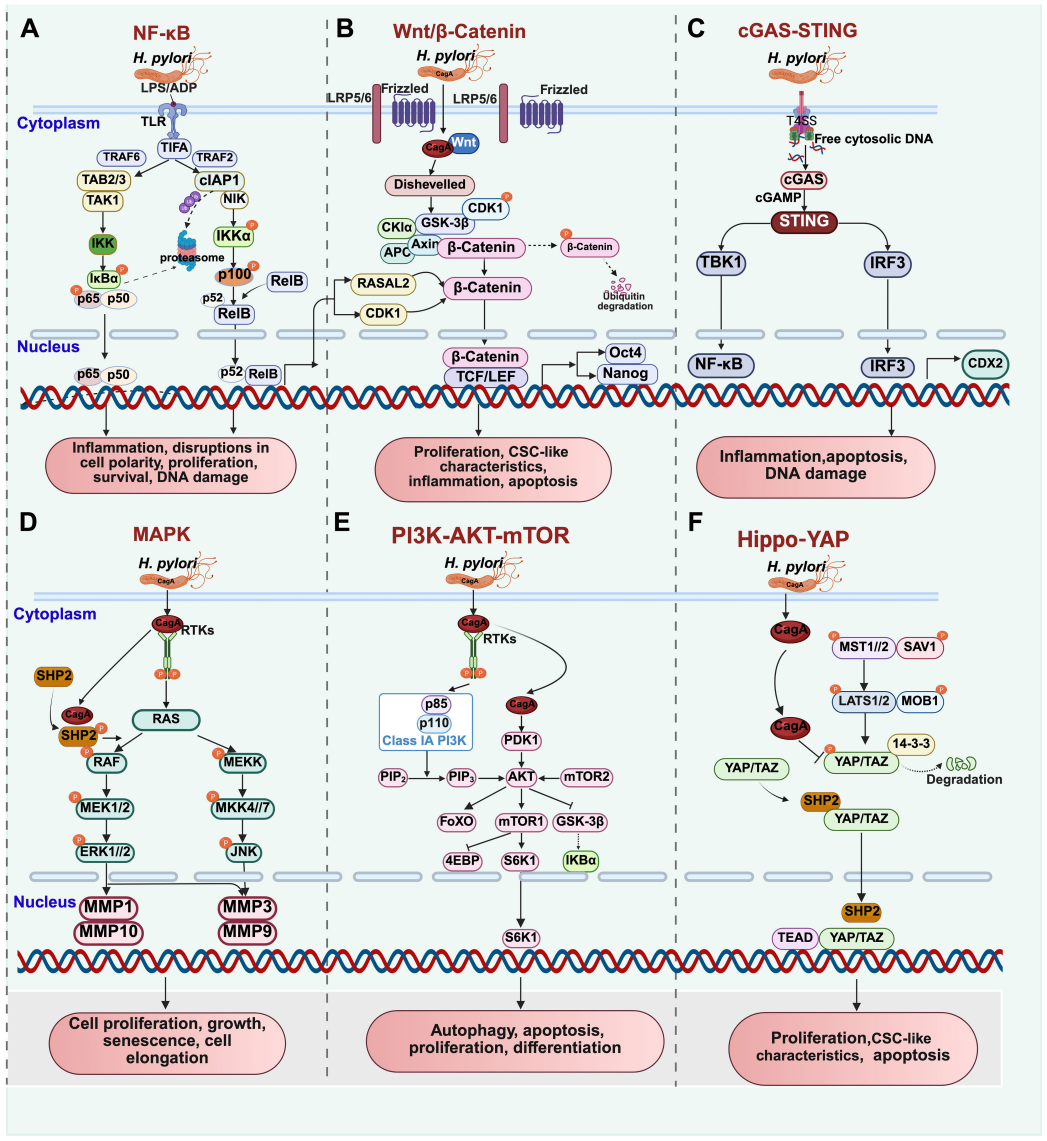
Molecular signal pathways related to *H. pylori*-induced gastric carcinogenesis. **(A)** NF-κB signaling pathway; **(B)** Wnt/β-Catenin signaling pathway; **(C)** cGAS-STING signaling pathway; **(D)** MAPK signaling pathway; **(E)** PI3K-AKT-mTOR signaling pathway. **(F)** Hippo-YAP signaling pathway.

Interaction with TRAF6 facilitates the recruitment of transforming growth factor-beta-activated kinase 1 (TAK1) through TAK1-binding proteins 2/3 (TAB2/3) and IKK complexes, resulting in IκBα degradation via phosphorylation. This process enables the nuclear translocation of RelA/p50, activating the canonical NF-κB pathway (Sokolova et al., 2018)(**Figure 1A**). Conversely, TIFA’s interaction with TRAF2 within the NIK (NF-κB-inducing kinase) regulatory complex (composed of TRAF2, TRAF3, and cIAP1) promotes NIK stabilization by triggering the degradation of cellular inhibitor of apoptosis 1 (cIAP1). The accumulation of NIK initiates IKKα phosphorylation, leading to p100 processing into p52 through phosphorylation and proteasomal cleavage. Consequently, the nuclear translocation of the p52/RelB dimer activates the non-canonical NF-κB pathway (Maubach et al., 2021) (**Figure 1A**).

Chronic gastritis has the potential to progress to gastric cancer, primarily through mechanisms involving CagA translocation that activates the NF-κB signaling pathway, leading to DNA damage and disruptions in cell polarity and proliferation (Yan et al., 2021). Research by Lin et al (Lin et al., 2019). indicates that MUC17 can impede *H. pylori* CagA translocation by downregulating NF-κB-mediated CEACAM1-3S expression in gastric epithelial cells.

In the context of *H. pylori* infection, the epigenetic downregulation of membrane-bound mucins (MUC) is observed. This downregulation amplifies CagA translocation, which in turn enhances the expression of DNA methyltransferase 1 (DNMT1) via the NF-κB signaling pathway (Zhang et al., 2016). Elevated DNMT1 levels promote the hypermethylation of tumor suppressor genes, fueling the progression of gastric cancer.

Further studies by Wang et al (Jia et al., 2022a). suggest that *H. pylori* induces the expression of differentiated and embryonic chondrocyte gene 1 (DEC1) by activating the Akt/NF-κB signaling pathway, with its expression positively correlated with gastric cancer progression. Additionally, the long non-coding RNA DLEU1 functions as an oncogene in gastric cancer, potentially induced by *H. pylori* infection. Its mechanism involves modulating the NF-κB signaling pathway, thereby contributing to the initiation and advancement of gastric tumors (Ghodrati et al., 2022).

High expression of guanine nucleotide-binding protein subunit beta-4 (GNB4) in gastric cancer (GC) patients is significantly associated with poor survival prognosis. *H. pylori* infection can activate the NF-κB signaling pathway, leading to the upregulation of TET1 expression. TET1 then binds to the demethylation-modified promoter region of GNB4, enhancing its expression and contributing to the unfavorable prognosis in patients (Liu et al., 2023). However, further research is needed to fully understand the expression and underlying molecular mechanisms of GNB4.

In addition to GNB4, certain genes play a role in promoting the development of precursor lesions to gastric cancer. For example, DARPP-32 is frequently amplified and overexpressed in cancer tissues. Upregulation of DARPP-32 expression following *H. pylori* infection activates NF-κB, promoting the progression of gastric cancer precursor lesions, ultimately leading to the development of gastric cancer (Zhu et al., 2017). Moreover, the NF-κB signal can be transmitted from PIEZO1, a mechanosensor, to the YAP1 signal known as a carcinogenic pathway. This transmission promotes intestinal metaplasia towards gastric cancer (Chen et al., 2023).

Furthermore, *H. pylori* has implications for the distant metastasis of gastric cancer. Wang et al. investigated the downregulation of microRNA-204 in gastric mucosal cells due to *H. pylori* infection. This downregulation led to increased expression of BIRC2, a target gene of microRNA-204, and enhanced activity in the BIRC2/TNF-α/NF-κB signaling pathway. These changes contributed to the angiogenesis and metastasis of gastric cancer cells (Chen et al., 2020).

### 2.2 Wnt/β-catenin signaling

The Wnt/β-catenin signaling pathway plays a crucial role in both embryonic development and adult tissue homeostasis, comprising two distinct types: the canonical and non-canonical Wnt pathways. Activation of the canonical Wnt/β-catenin signaling pathway depends on the interaction between extracellular Wnt ligands, seven-transmembrane receptor Frizzled proteins (Fzd), and their co-receptor low-density lipoprotein receptor-related protein (LRP). This interaction triggers the recruitment of the scaffold protein Disheveled (Dvl), leading to the phosphorylation of the co-receptor LRP5/6 (Yu et al., 2021; Liu et al., 2022). Subsequently, Wnt signaling disrupts the destruction complex, consisting of the scaffold protein Axin, tumor suppressor gene product Adenomatous Polyposis Coli (APC), Lactase kinase 1 (CK1), and GSK3β, preventing the phosphorylation of β-catenin (Schaefer and Peifer, 2019). The β-catenin then translocates into the nucleus to regulate transcription by binding to T cell factor (TCF)/lymphoid enhancer factor (LEF), thereby maintaining low levels of cytoplasmic β-catenin (Schunk et al., 2021).

When gastric epithelial cells (GECs) are infected with CagA-positive *H. pylori*, the destruction complex of the Wnt signaling pathway is inactivated, preventing the phosphorylation of cytoplasmic β-catenin (Franco et al., 2005). Studies have shown that *H. pylori* enhances the nuclear accumulation and transcriptional activation of Wnt in a CagA-dependent manner, resulting in an increase in Nanog and Oct4 expression, specific cancer stem cell (CSC) markers. This process promotes CSC-like characteristics in gastric cancer (GC) cells (Yong et al., 2016). *H. pylori* infection also increases the presence of Low-Density Lipoprotein Receptor-Related Protein 8 (LRP8) within CSCs, facilitating the nuclear translocation and transcriptional activity of β-catenin, ultimately promoting GC development. This mechanism can involve inhibiting the binding of E-cadherin and β-catenin, and promoting the formation of a CagA/LRP8/β-catenin complex (Liu et al., 2024).

Previous research from our team has shown that *H. pylori* infection induces gastritis by upregulating transcription factors ASCL1/AQP5, activating the Wnt/β-catenin signaling pathway, and subsequently triggering apoptosis and inflammation in gastric epithelial cells (Zuo et al., 2022). Interaction with T-cell factor/lymphoid enhancer factor (TCF/LEF) family transcription factors causes β-catenin to initiate Wnt-dependent gene expression (Koelman et al., 2022). This leads to the upregulation of cancer-related genes like cyclin D1, cyclin E1, and c-Myc, influencing cellular processes such as differentiation, proliferation, migration, and adhesion, ultimately contributing to tumorigenesis (Nagy et al., 2011; Silva-Garcia et al., 2019).

Furthermore, *H. pylori* elevates the expression of RAS protein activator like 2 (RASAL2) through the NF-κB signaling pathway. NF-κB promotes GC progression by increasing the levels of nuclear β-catenin through a novel NF-κB/RASAL2/β-catenin signaling axis (Cao et al., 2022) (**Figure 1B**). Zhu et al. have confirmed that the activation of the NF-κB signaling pathway induces the expression of cyclin-dependent kinase 1 (CDK1), which inhibits GSK-3β activity by direct binding, leading to the accumulation and activation of β-catenin. Interestingly, these effects can be reversed by CDK1 inhibitors or silencing CDK1 (Zhu et al., 2023).

### 2.3 cGAS-STING signaling

In mammals, the cyclic GMP-AMP (cGAMP)-stimulator of interferon genes (STING) signaling axis represents a critical innate immune pathway that plays a significant role in inducing DNA replication stress, genome instability, and impacting tumor initiation, progression, and metastasis (Samson and Ablasser, 2022). Within this pathway, cGAMP synthase (cGAS) functions as a crucial enzyme, serving as a DNA sensor that detects cytosolic DNA signals (Sun et al., 2013). When DNA ligands bind to cGAS, it catalyzes the conversion of ATP and GTP into the cyclic dinucleotide 2’,3’-cGAMP (cGAMP). This signaling molecule then activates STING, leading to the recruitment of TBK1, phosphorylation and activation of the transcription factor IRF3, and subsequent induction of type I interferon (IFN) responses (Jiao et al., 2024). Additionally, activation of the cGAS-STING pathway by cytoplasmic chromatin fragments can trigger cellular autophagy and senescence, further highlighting the diverse effects and regulatory mechanisms of this innate immune pathway (Li and Chen, 2018; Nassour et al., 2019).

Upon infection of gastric epithelial cells by *H. pylori*, the initial components of the innate immune system, such as epithelial cells, macrophages, and dendritic cells, are disrupted, leading to a dysregulation of signaling pathways that can impact tumor initiation (Zavros and Merchant, 2022). Recent studies indicate that *H. pylori* activates STING signaling pathways by translocating DNA into epithelial cells via the Type IV secretion system (T4SS). This DNA translocation, coupled with the activation of IRF3, may represent *H. pylori* ‘s regulatory mechanism for modulating the initial innate immune response and sustaining the long-term activation of inflammatory pathways (Dooyema et al., 2022).

Furthermore, research has shown that *H. pylori* triggers the cGAS-STING pathway, resulting in the activation of IRF3 transcriptional activity. This activation leads to the upregulation of caudal-type homeobox 2 (CDX2) and mucin 2 (MUC2), promoting intestinal metaplasia (Liang et al., 2023) (**Figure 1C**). Additionally, the inactivation of the YAP/TAZ signaling pathway by *H. pylori* infection inhibits transcriptional activity, causing a loss of nuclear membrane integrity. This loss exposes DNA, which is then recognized and activated by cGAS, initiating the STING pathway and subsequent signaling cascade (Sladitschek-Martens et al., 2022).

### 2.4 MAPK signaling

The mitogen-activated protein kinases (MAPK) signaling cascade involves three primary kinases: MAPK, MAPK kinase (MAPKK), and MAPKK kinase (MAPKKK). These kinases regulate various cellular processes such as proliferation, differentiation, apoptosis, and stress responses by phosphorylating downstream molecules (Jiang et al., 2022). MAPK comprises three major subfamilies: extracellular signal-regulated kinases (ERK MAPK, Ras/Raf/MEK/ERK), c-jun N-terminal kinases or stress-activated protein kinases (JNK or SAPK), and MAPK14 (p38-α) (Fang and Richardson, 2005).

Among these subfamilies, the canonical Ras/Raf/MEK/ERK pathway plays a crucial role in cell proliferation and is involved in events like occurrence, development, and carcinogenesis. This pathway is essential for both intercellular and intracellular signaling (Morgos et al., 2024). Key MAPK pathways in mammalian cells include the MEK1/2→ERK1/2 pathway, which is commonly associated with cell proliferation and cancer progression, with Ras/Raf proteins acting as upstream signals (Lucas et al., 2022). Additionally, the MKK3/6→p38 pathway regulates inflammatory responses (Romero-Becerra et al., 2022), while the MKK4/7→JNK pathway governs cell apoptosis, motility, metabolism, and other disease pathogenesis (Lee et al., 2021).

The Src homology 2 (SH2)-containing protein tyrosine phosphatase-2 (SHP2) located on the plasma membrane responds to CagA released by *H. pylori*, leading to its recruitment and activation. Subsequently, SHP2 activates the Ras/Raf/MEK/ERK signaling pathway, resulting in cell elongation to form filopodia and inducing morphological changes known as the “hummingbird phenotype” and cell scattering (Higashi et al., 2004; Belogolova et al., 2013). This activation can lead to the downregulation of downstream tumor suppressor genes like Gastrokine1 (GKN1) and Runt-related transcription factor 3 (Runx3), ultimately promoting abnormal proliferation and invasion in gastric cancer (Chung Nien Chin et al., 2020; Song et al., 2022).

Additionally, the *H. pylori* cell wall component lipopolysaccharide (LPS) can also contribute to the activation of the MAPK signaling pathway. By releasing inflammatory factors, LPS activates the p38 MAPK and JNK pathways, further influencing cellular responses and potentially contributing to disease progression (Xu et al., 2018; Ji et al., 2023).

Moreover, the adenosine A2B receptor (A2BR) has been shown to promote *H. pylori* -induced gastric ulcers by activating the p38 MAPK pathway (Tang et al., 2023). Experimental studies have revealed that mitogen-activated protein kinase 6 (MAP2K6) is a direct target of miR-1298-5p, influencing the survival and invasion abilities of gastric cancer cells through autophagy regulation. This mechanism contributes to gastric cancer development via the MAP2K6/p38 MAPK axis (Li et al., 2022).

*H. pylori* infection also leads to the upregulation of heparinase (HPA) in gastric inflammation, facilitating extracellular matrix remodeling and worsening inflammatory infection. HPA plays a role in a context driven by enhanced NF-κB and p38-MAPK signaling, thereby promoting gastric cancer development and progression. These interactions highlight the intricate molecular mechanisms underlying the impact of *H. pylori* infection on gastric pathophysiology and tumorigenesis (Liu et al., 2018; Tang et al., 2021).

Additionally, MAPK plays a crucial role in promoting gastric cancer metastasis by influencing various biological functions. Matrix metalloproteinases (MMPs) are key molecules involved in cancer invasion and metastasis as they can degrade the extracellular matrix and intercellular adhesion molecules. Upon CagA release, there is an upregulation of MMP-3 and MMP-9 *in vivo*, primarily mediated by the ERK1/2 and JNK pathways (Karayiannis et al., 2023).

The *H. pylori* cytotoxin-associated gene pathogenicity island (cagPAI), which is linked to gastric cancer metastasis, induces the upregulation of MMP-1 and MMP-10 expression through the activation of the ERK1/2 signaling pathway. These molecular mechanisms highlight how *H. pylori* infection can impact MMP expression levels via MAPK signaling pathways, contributing to gastric cancer progression and metastasis (Jiang et al., 2014) (**Figure 1D**).

### 2.5 PI3K/AKT/mTOR signaling pathway

The PI3K-Akt-mTOR signaling pathway is essential for regulating fundamental cellular activities. Phosphoinositide 3-kinase (PI3K) can be categorized into three different kinase classes based on their functional and structural characteristics, participating in the phosphorylation of inositol ring 3’-OH groups in phospholipids (Tewari et al., 2022). Class IA PI3K is particularly important for cell proliferation and activation, comprising regulatory subunits (p85α/p55α/p50α, p85β, or p55γ) (Shorning et al., 2020) and catalytic subunits (p110, p110α, p110β, or p110δ) that form heterodimers (Shorning et al., 2020; Xu et al., 2020).

Upon binding to receptor tyrosine kinases (RTKs) or G protein-coupled receptors (GPCRs), PI3K converts its substrate phosphatidylinositol (4,5)-bisphosphate (PIP2) to phosphatidylinositol (3,4,5)-trisphosphate (PIP3), leading to the rapid production of PI3P (Fresno Vara et al., 2004). PIP3 then recruits other kinases like phosphoinositide-dependent kinase 1 (PDK1) and AKT, both containing pleckstrin homology (PH) domains crucial for PI3K signal transduction (Fresno Vara et al., 2004). AKT has three subtypes (AKT1/PKBa, AKT2/PKBβ, and AKT3/PKBγ), each with distinct domains involved in phosphorylation and regulation (Nepstad et al., 2020).

Phosphorylated AKT activates numerous downstream targets, including mTOR, thereby promoting protein synthesis, cell growth, survival, and movement (Glaviano et al., 2023). The mammalian target of rapamycin complexes (mTORC), specifically mTORC2, interacts with PDK1 to activate AKT through phosphorylation, highlighting the interconnected nature of this signaling pathway in regulating various cellular functions.

*H. pylori* infection triggers the activation of the PI3K/AKT/mTOR pathway in gastric epithelial cells, leading to significant changes in processes such as apoptosis, proliferation, and differentiation. This activation contributes to the transformation of epithelial cells into tumor cells (Wang et al., 2021; Glaviano et al., 2023). The activated mTORC1 regulates crucial cellular functions, including protein synthesis, ribosome biogenesis, mRNA transcription, autophagy, and cell growth, by phosphorylating eukaryotic initiation factor 4E-binding protein 1 (4E-BP1) and p70 ribosomal protein S6 kinase (S6K) (Porta et al., 2014; Napolitano et al., 2020).

The PI3K/AKT/mTOR signaling pathway plays a vital role in cell survival by inhibiting apoptosis-related genes like Bcl-2-associated death promoter (BAD), Bcl-2-associated X (BAX), caspase-9, GSK-3, and Forkhead box protein O (FoxO). Notably, glycogen synthase kinase-3β (GSK-3β) is a significant downstream component (Borden et al., 2021; Wang et al., 2024). Upon *H. pylori* infection, the activation of the PI3K/AKT/mTOR pathway leads to GSK-3β inactivation, promoting crosstalk between the Wnt/β-catenin and NF-κB signaling pathways. This interaction results in the upregulation of downstream genes, inducing pro-inflammatory responses and contributing to gastric carcinogenesis (Lin et al., 2020) (**Figure 1E**).

The increased release of pro-inflammatory cytokines can elevate free radical production, leading to DNA methylation and potentially causing mutations in genes such as AKT1/AKT2/AKT3. Additionally, CagA promotes autophagy, enhances the expression of downstream inflammatory cytokines through the C-met/Akt signaling pathway, and aids in the progression of gastric cancer (Li et al., 2017a). The PI3K/Akt signaling pathway further drives gastric cancer progression by promoting epithelial-mesenchymal transition (EMT). These complex interactions underscore the critical role of the PI3K/AKT/mTOR pathway in *H. pylori* -related gastric carcinogenesis and disease progression (Chi et al., 2022).

### 2.6 Hippo signaling

The Hippo signaling pathway, first identified in fruit fly tissues and evolutionarily conserved, plays a critical role in regulating cell proliferation, apoptosis, stem cell pluripotency, and tumorigenesis (Chen et al., 2019). In mammals, this pathway involves core kinase cascades, including Sav1 protein (WW45), MOB kinase activator 1A/B (MOB1A/B), Mammalian sterile 20-like 1/2 (MST1/2), Large tumor suppressor homolog 1/2 (LATS1/2), and downstream effectors with WW domains (YAP and TAZ) (Moldovan et al., 2024; Wesener et al., 2024).

Upon activation of the Hippo signaling pathway by external stimuli from the cellular microenvironment, upstream kinases MST1/2 interact with Sav1 and phosphorylate LATS1/2. Activated LATS1 directly phosphorylates YAP, leading to YAP binding to 14-3–3 proteins and subsequent cytoplasmic sequestration, preventing tissue overgrowth (Hansen et al., 2015). YAP/TAZ are crucial for maintaining a balance in stem cell niches, promoting induced pluripotent stem cell (iPSC) generation, and mediating regeneration and cancer initiation signals (Yagi et al., 2024).

Furthermore, LATS1/2 phosphorylates YAP, facilitating the recruitment of E3 ubiquitin ligase SCFb-TrCP, leading to YAP ubiquitination and degradation, thereby controlling intracellular YAP levels (Zhao et al., 2010; Kim et al., 2013). However, in cases where the Hippo signaling pathway is inactive, unphosphorylated YAP translocates from the cytoplasm to the nucleus in conjunction with the transcription factor TEADs, exhibiting oncogenic properties. Concurrently, activating downstream oncogenes like CYR61, CTGF, and cyclin D1 transcription can change the characteristics of the extracellular matrix. This may lead to the recruitment of cancer-associated fibroblasts, thereby altering the GC microenvironment and promoting tumor progression (Li et al., 2017b; Holden and Cunningham, 2018). The intricate regulation of the Hippo signaling pathway highlights its significance in cellular homeostasis and disease pathogenesis, including cancer development.

A recent study has revealed that *H. pylori* infection of gastric epithelial cells (GECs) leads to an increase in integrin-linked kinase (ILK) levels. This rise in ILK inhibits the Hippo signaling pathway, resulting in the translocation of YAP into the nucleus and promoting gastric cancer progression (Li et al., 2024). Previous research indicated higher YAP expression levels in chronic gastritis tissues of *H. pylori*-positive patients compared to those who tested negative (Li et al., 2018). These findings suggest that activating the YAP signaling pathway may be a primary molecular mechanism through which *H. pylori* promotes gastric carcinogenesis.

Further investigations supported that *H. pylori* infection triggers the activation of YAP and β-catenin, which cooperatively target downstream genes like CDX2, LGR5, and RUVBL1 to drive gastric mucosal lesions (Li N. et al., 2023). This cascade ultimately leads to the invasion and migration of gastric adenocarcinoma cells, facilitating carcinogenic epithelial-to-mesenchymal transition (EMT) (Li et al., 2018). Additionally, the YAP homolog TAZ establishes a positive feedback loop with β-catenin, enhancing *H. pylori*-induced gastric mucosal carcinogenesis (Xu et al., 2022).

The key player in this process is the cytotoxin-associated gene A (CagA) secreted by *H. pylori*, which augments its activity by binding to SHP2 (Watkins et al., 2022). The interaction between non-phosphorylated YAP and TAZ with SHP2 enhances their nuclear localization, activating TEAD-regulated genes (**Figure 1F**). This activation further drives abnormal proliferation and differentiation of gastric epithelial cells (Tsutsumi et al., 2013).

Collectively, these findings shed light on the intricate molecular mechanisms orchestrated by *H. pylori* to promote gastric carcinogenesis. This is achieved through dysregulation of the Hippo-YAP/TAZ pathway and interactions with downstream effectors like CagA and SHP2.

## 3 Alternative signaling pathway modulated by *H. pylori* infection

### 3.1 Sonic-Hedgehog pathway

The Hedgehog/GLI (HH/GLI) signaling pathway serves as a critical regulator of gastric epithelial cell homeostasis, governing both physiological processes (proliferation and differentiation) (Merchant, 2012) and pathological conditions (inflammation and carcinogenesis) during *H. pylori* infection (Katoh and Katoh, 2005). This evolutionarily conserved pathway comprises three Hedgehog ligands (SHH, IHH, DHH)), the transmembrane HH receptor patch 1 (PTCH1), the signal transducer Smoothened (SMO), the negative regulator SUFU, and downstream transcription factors (GLI2, GLI3). In the basal state, unbound PTCH1 maintains SMO inhibition, while SUFU sequesters GLI2/3 in the cytoplasm, maintaining them in a phosphorylated, inactive form. Upon *H. pylori* infection, SHH binding to PTCH1 relieves SMO inhibition, triggering GLI2/3 nuclear translocation and subsequent activation of oncogenic target genes. Recent studies demonstrate the pathway’s clinical relevance through multiple mechanisms: (1) Vivien et al. revealed that PI3K/AKT/mTOR -mediated GLI2 upregulation induces PD-L1 expression, facilitating immune evasion (Koh et al., 2021); (2) Zhu et al. established that chemotherapy-resistant, EMT-type gastric cancers exhibit GLI2 overexpression, and that GLI2 knockdown restores cisplatin sensitivity. These findings position HH/GLI inhibition as a promising therapeutic strategy, particularly for treatment-resistant gastric cancer subtypes (Zhu et al., 2025).

### 3.2 Type I Interferon pathway

The canonical Type I Interferon (IFN-I) pathway consists of IFNα receptor (IFNAR), Janus kinase 1(JAK1), tyrosine kinase 2 (TYK2), signal transducer and activator of transcription proteins (STAT1/STAT2), and interferon regulatory factor 9 (IRF9), which are ubiquitously expressed in most cell types (Ivashkiv and Donlin, 2014). This pathway is primarily activated by IFN-α and IFN-β. During *H. pylori* infection, bacterial RNA is detected by TLR8 through pathogen-associated molecular patterns (PAMPs), triggering downstream NF-KB or IRF signaling pathways that induce IFN expression (Gantier et al., 2010; Lee et al., 2022).

Recent studies have elucidated the complex role of IFN-I signaling in gastric carcinogenesis: (1) Xiang et al. demonstrated that IFN-α secreted by plasmacytoid dendritic cells (pDCs) promotes the generation of Schlafen 4-expressing myeloid derived suppressor cells (SLFN4+MDSCs), creating an immunosuppressive tumor microenvironment that facilitates intestinal metaplasia and gastric cancer transformation (Xiang et al., 2021); (2) Tomohiro et al. revealed that *H. pylori* infection activates nucleoside binding oligomerization domain 1 (NOD1) to induce protective IFN-γ responses, with IFN-γ-deficient mice showing significantly increased bacterial loads (Watanabe et al., 2011); and (3) Activation of the TRIF-IFN-I signaling pathway initiates a cascade of inflammatory responses that ultimately contribute to gastric carcinogenesis. Through induction of interferon-stimulated genes (ISGs), these IFN-mediated pathways modulate both antimicrobial defense and tumor-promoting inflammation, highlighting their dual role in *H. pylori* pathogenesis (Bali et al., 2024).

## 4 Crosstalk among *H. pylori*-associated signaling pathways

**Figure 2:**
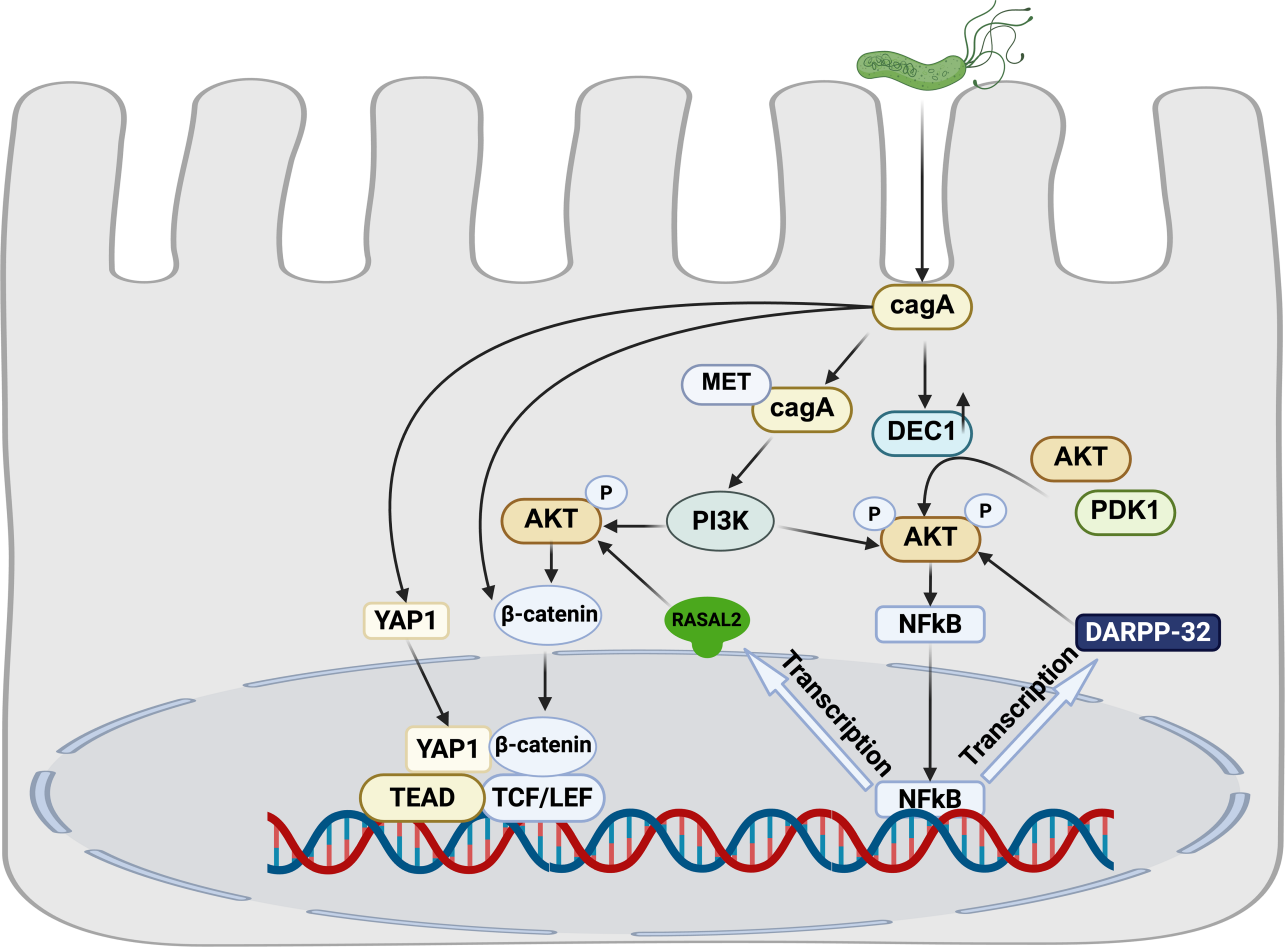
Crosstalk among *H. pylori*-associated signaling pathways.

The oncogenic effects linked to *H. pylori* infection are not solely attributed to a single pathway but rather arise from the complex interplay of multiple signaling cascades (**Figure 2**) (Alipour, 2021; Kashyap et al., 2023).

### 4.1 PI3K/AKT, NF-κB and Wnt/β-catenin signaling pathway

*H. pylori* infection upregulates dopamine- and cAMP-regulated phosphoprotein (DARPP-32), a known transcriptional target of NF-κB. This upregulation activates the pro-survival AKT signaling pathway, mechanistically contributing to gastric tumorigenesis (Zhu et al., 2017).Furthermore, *H. pylori* infection induces expression of DEC1, which subsequently activates the Akt/NF-κB signaling axis. This activation cascade ultimately promotes accelerated proliferation of gastric epithelial cells, representing an additional pathway through which H. pylori infection drives oncogenic progression (Jia et al., 2022b).

The CagA oncoprotein, a key virulence factor of *H. pylori*, serves as a critical molecular hub that coordinately regulates the PI3K/AKT, NF-κB and Wnt/β-catenin signaling cascades. Following T4SS-mediated delivery into gastric epithelial cells, non-phosphorylated CagA interacts with activated Met, inducing sustained PI3K/Akt pathway activation. This persistent signaling subsequently activates both β-catenin and NF-κB downstream effectors (Suzuki et al., 2009). Mechanistically, we identified RASAL2 as a direct transcription target of NF-κB. Activated RASAL2 further amplifies oncogenic signaling by promoting nuclear β-catenin translocation through the Akt/β-catenin axis (Cao et al., 2022). Clinical correlation analyses demonstrate that RASAL2 overexpression significantly correlates with poor prognosis and chemotherapy resistance in GC patients.

### 4.2 Hippo and Wnt/β-catenin signaling pathway

The Hippo and Wnt signaling pathways engage in extensive functional crosstalk, playing pivotal roles in governing cell proliferation, stem cell self-renewal, and tissue homeostasis (Li et al., 2019). Mechanistically, emerging evidence demonstrates that YAP/TAZ, the core transcriptional effectors of the Hippo pathway, physically interact with β-catenin to modulate its protein stability (Azzolin et al., 2014). Our experimental data demonstrate that H. pylori infection triggers coordinated concurrent nuclear translocation of both YAP/TAZ and β-catenin. This synergistic nuclear accumulation promotes oncogenic phenotypes, including enhanced proliferation, invasion and migration of in gastric epithelial cells. Notably, pharmacological inhibition of either YAP or β-catenin significantly ameliorates H. pylori-induced gastric pathology in our murine model studies (Li N. et al., 2023).

## 5 Exploiting the host DNA damage response

DNA damage refers to physical or chemical alterations in cellular DNA, which can occur when cells are exposed to various damaging stimuli like ionizing radiation, chemicals, free radicals, and topological changes (Li Q. et al., 2023). These alterations have the potential to disrupt the storage and transmission of genetic information.

*H. pylori* infection can lead to DNA damage in host cells through both direct and indirect mechanisms, such as oxidative damage and double-strand breaks (DSBs), while also impeding the cell’s ability to repair DNA damage (Xu et al., 2025). The consequences of this DNA damage may include increased genetic instability, the progressive accumulation of oxidative DNA damage in specific genes (e.g., p53), activation of oncogenes, inactivation of tumor suppressor genes, and ultimately the promotion of gastric cancer development (Murata-Kamiya and Hatakeyama, 2022).

Moreover, *H. pylori* infection induces DNA damage in host cells, resulting in genomic instability, which manifests as microsatellite instability (MSI), chromosomal instability (CIN), and abnormal activation of telomerase (Hudler, 2012). These cumulative effects underscore the role of *H. pylori* in triggering DNA damage-related pathways that contribute to the pathogenesis of gastric cancer. Further understanding of these processes is crucial for developing targeted therapeutic interventions aimed at mitigating the adverse effects of *H. pylori*-induced DNA damage on host cells.

*H. pylori* infection has been shown to increase oxidative stress in gastric epithelial cells (Han et al., 2022). The bacterial effector protein CagA derived from *H. pylori* stimulates the expression of spermine oxidase (SMOX). SMOX, in turn, generates reactive oxygen species (ROS) (Dzikowiec et al., 2024), leading to DNA damage and oxidative stress. This process not only exacerbates the carcinogenic effects of *H. pylori* but also activates inflammation and β-catenin signaling pathways (Li N. et al., 2023).

The excessive production of ROS and reactive nitrogen species (RNS) elicited by *H. pylori* infection results in various forms of DNA damage, including point mutations, DNA adducts, and single or double-strand DNA breaks (DSBs) (Guo et al., 2023). Notably, 8-hydroxy-2’-deoxyguanosine (8-OHdG), a prominent oxidative modification product of DNA, is significantly expressed in gastric cancer tissues. The accumulation of 8-OHdG due to ROS leads to DNA damage, underscoring the role of oxidative stress in gastric carcinogenesis (Nasif et al., 2022).

**Figure 3:**
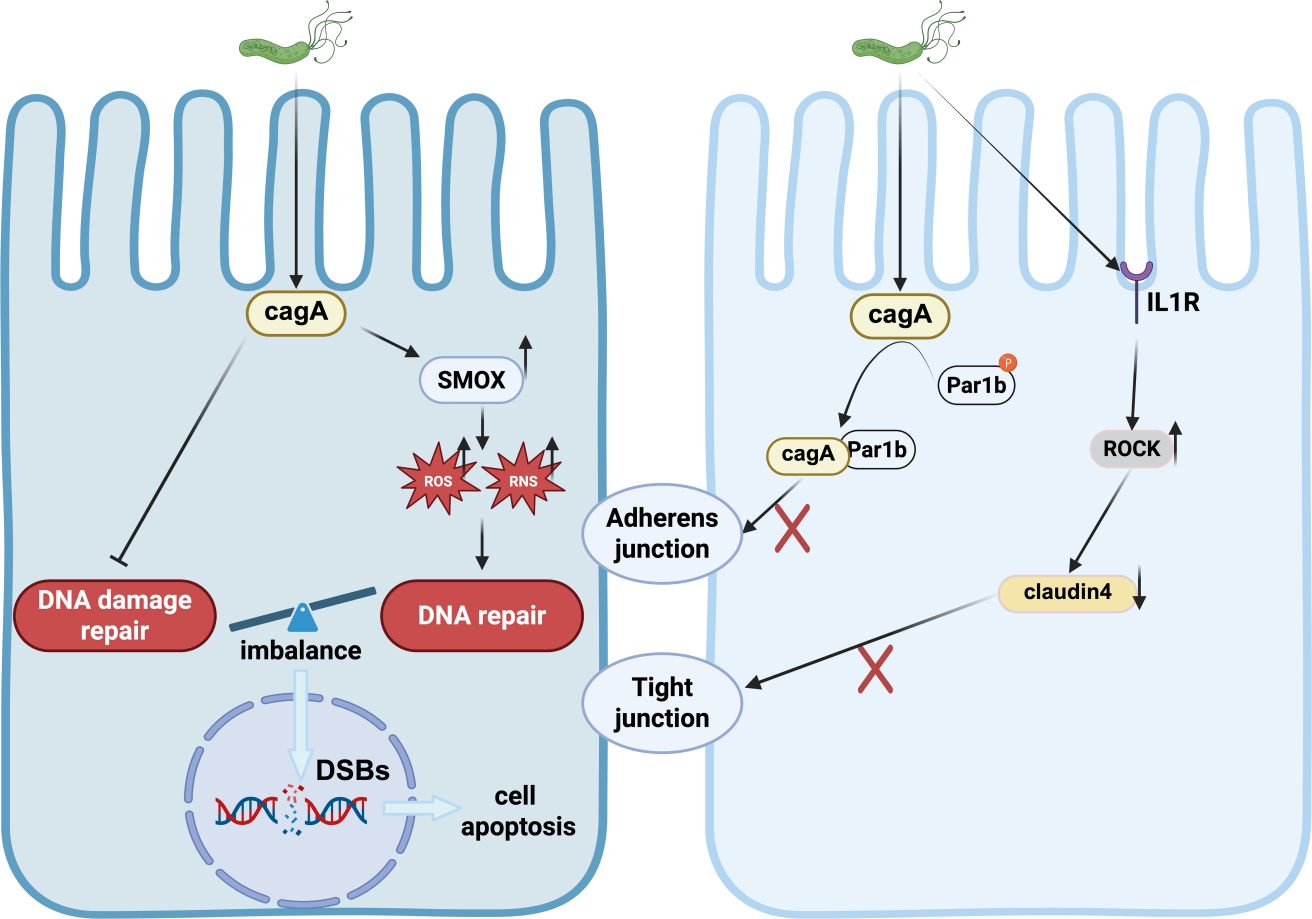
DNA damage and Disruption of tight junction induced by *H.pylori*.

Apurinic/apyrimidinic endonuclease/redox factor 1 (APE1) plays a crucial role in cellular response to oxidative stress. While *H. pylori*-induced oxidative stress initially increases APE1 levels and aids in DNA damage repair, chronic *H. pylori* infection may suppress APE1 expression over time (**Figure 3**). This suppression can impair DNA damage repair mechanisms, leading to genetic instability and contributing to the development and progression of gastric cancer (Kontizas et al., 2020; Rios-Covian et al., 2023).

The intricate interplay between *H. pylori* infection, oxidative stress, DNA damage, and repair mechanisms highlights the multifaceted impact of bacterial colonization on the molecular processes underlying gastric carcinogenesis. Understanding these complex interactions is essential for developing targeted therapeutic strategies to mitigate the adverse effects of *H. pylori*-induced oxidative stress and DNA damage in gastric epithelial cells.

## 6 Disruption of tight junction

Tight junctions play a pivotal role in maintaining the integrity of the epithelial barrier, which acts as a crucial boundary separating the internal and external environments of the body. These junctions create a continuous seal around cells, forming a physical barrier that restricts the passage of solutes and water through the paracellular space (O’Leary et al., 2021). The establishment and preservation of a fully functional epithelial barrier require the presence of polarized epithelial cells with distinct structural domains, including apical, basal lateral, and ciliated surfaces. Apart from well-coordinated intracellular cytoskeleton dynamics and interactions with the extracellular matrix (ECM), adhesion between neighboring epithelial cells is also essential for barrier function. This intercellular adhesion involves tight junctions (TJs) and adherens junctions (AJs) (Rodriguez-Boulan and Macara, 2014; Sumigray and Lechler, 2015), which work in concert to maintain cell-cell interactions and strengthen the epithelial barrier. Tight junctions provide a sealing function by forming a belt-like structure at the apical end of epithelial cells, while adherens junctions facilitate cell-cell adhesion through interactions with the actin cytoskeleton.

Collectively, the coordinated actions of tight junctions and adherens junctions are crucial for preserving epithelial barrier integrity, regulating paracellular permeability, and ensuring selective transport of molecules across epithelial layers. Disruption of these junctional complexes can compromise barrier function and contribute to various pathological conditions characterized by increased permeability and impaired epithelial integrity.

*H. pylori* infection is linked to structural alterations in epithelial cells that result in the depolarization of the epithelium. The bacterial effector protein, CagA, plays a key role in disrupting tight junctions (TJs). CagA binds to tight junction proteins such as zonula occludens-1 (ZO-1) and junctional adhesion molecule (JAM), targeting them to apical cell connections. This interaction is sufficient to disrupt apical junctions, leading to disturbances in epithelial cell differentiation, loss of polarity, and intercellular adhesion, ultimately enhancing cellular invasiveness (Amieva et al., 2003; Hurtado-Monzon et al., 2024).

Furthermore, CagA inhibits the kinase activity of partitioning-defective 1 (PAR1) and interferes with atypical protein kinase C (PKCα)-mediated PAR1 phosphorylation, resulting in junctional and polarity defects (Buckley and St Johnston, 2022). *H. pylori* infection promotes an abnormal interaction between cortical actin/Par1b/ZO-1 in tight junctions in a CagA-dependent manner, contributing to the disruption of gastric epithelial cell polarity (Sharafutdinov et al., 2024). Moreover, *H. pylori* disrupts the structure of the gastric epithelial barrier by activating IL-1 receptor type I (IL-1RI)-dependent Rho kinase (ROCK), which subsequently mediates the degradation of tight junction protein-4 within the tight junction complex (Lapointe et al., 2010). These tight junction proteins are essential components of the defense mechanism in gastric epithelial cells (**Figure 3**).

Studies conducted by Choi and colleagues have investigated the expression levels of TJP1 in *H. pylori*-positive gastric cancer patients and post-*H. pylori* eradication. Their findings suggest that TJP1 plays a significant role in gastric cancer development, highlighting the intricate relationship between *H. pylori*-induced alterations in tight junctions and the progression of gastric carcinogenesis (Choi et al., 2022). Understanding these mechanisms is critical for developing targeted interventions aimed at preserving epithelial integrity and mitigating the adverse effects of *H. pylori* infection on epithelial polarity and barrier function.

## 7 Conclusions and outlook

Gastric cancer (GC) is a complex malignancy that evolves gradually through a multi-step histopathological cascade. Infection with *H. pylori* plays a pivotal role in the initiation and progression of GC, serving as a crucial trigger in this process. The synthesis of arguments presented in this review underscores that disrupting essential cellular signaling pathways and inducing pathological changes in host cells are central to the carcinogenicity of CagA, a key virulence factor of *H. pylori*.

The impact of CagA on host cells results in aberrant signaling pathways that contribute to various changes, including but not limited to: (1) Immune Imbalance and Inflammatory Responses: CagA promotes immune imbalance and exacerbates inflammatory responses, leading to chronic inflammation in gastric epithelial cells. This persistent inflammation contributes to the development of adenomas and ultimately gastric cancer. (2) Genomic Instability: Production of toxic substances by CagA induces high-frequency mutations in the cell genome, resulting in accumulated genomic instability. These mutations alter genetic material, facilitating tumorigenesis. (3) Cell Cycle Dysregulation: Abnormal regulation of the cell cycle by CagA leads to the release of continuous proliferative signals, increasing the risk of cellular carcinogenesis. (4) Disruption of Polarity Regulation: CagA disrupts the function of polarity-regulating proteins, undermining cellular function and tissue integrity, ultimately promoting tumor formation. The development of targeted drugs specifically designed for these biomolecules is anticipated to enhance clinical efficacy and increase drug sensitivity in GC patients. This advancement holds promise as a prospective clinical treatment strategy.

Several molecularly targeted therapies are currently under preclinical or clinical investigation, including: (1) HER2-directed therapy, where overexpression drives oncogenesis progression through PI3K/AKT and MAPK pathway activation, making trastuzumab the standard first-line treatment of HER2-positive GC (Sierra et al., 2018); (2) PI3K/AKT pathway inhibition, as evidenced by LY294002’s ability to suppress p-AKT (a well-established marker of tumor progression and poor prognosis) and improve clinical outcomes (Kobayashi et al., 2006); and (3) CTGF targeting, which promotes invasion and metastasis through E-cadherin downregulation, NF-KB activation, and β-catenin-mediated EMT (Shen et al., 2021), with siRNA-mediated CTGF blockade showing significant anti-metastatic effects in preclinical models. These findings highlight promising therapeutic avenues for GC treatment. These research findings systematically elucidate the molecular pathway mechanisms through which *H. pylori* induces the transformation of gastric epithelial cells into gastric cancer. Understanding these intricate processes is essential for developing targeted therapeutic strategies aimed at mitigating the adverse effects of *H. pylori* infection on gastric epithelial cells and potentially preventing the development of gastric cancer.
